# COMMON INFECTIONS IN RELATION TO SLEEP DISTURBANCES BY RACE IN A POPULATION-BASED COHORT OF ADULTS

**DOI:** 10.1093/geroni/igad104.2991

**Published:** 2023-12-21

**Authors:** Jill Rabinowitz, Yiwei Yue, Diefei Chen, Robert Yolken, Chandra Jackson, Brion Maher, William Eaton, Adam Spira

**Affiliations:** Johns Hopkins Bloomberg School of Public Health, Baltimore, Maryland, United States; Johns Hopkins Bloomberg School of Public Health, Baltimore, Maryland, United States; Johns Hopkins, Baltimore, Maryland, United States; Johns Hopkins School of Medicine, Baltimore, Maryland, United States; National Institute of Environmental Health Sciences, Research Triangle Park, North Carolina, United States; Johns Hopkins Bloomberg School of Public Health, Baltimore, Maryland, United States; Johns Hopkins Bloomberg School of Public Health, Baltimore, Maryland, United States; Johns Hopkins Bloomberg School of Public Health, Baltimore, Maryland, United States

## Abstract

Common infections (e.g., human herpesviruses and Toxoplasma gondii (TOX)), are associated with poor health outcomes, but less is known about associations between these infections and sleep. Due to, for instance, structural racism leading to policies and practices resulting in relative economic disadvantage and subsequently poorer living condition, sleep disturbances and common infections are more prevalent in minoritized populations. We investigated the cross-sectional associations of common infections (i.e., herpes simplex virus type 1 (HSV-1), cytomegalovirus (CMV), varicella zoster virus (VZV), Epstein-Barr virus (EBV), and TOX with sleep disturbances, and determined whether these associations differed by race in 602 adults enrolled in the Baltimore Epidemiologic Catchment Area Study (mean age=59.0±12.8 years, 36.9% male, 35.6% minoritized). Participants donated a blood sample and were asked whether they had the insomnia symptoms “trouble falling asleep, staying asleep, or waking up too early” and reported on their habitual sleep duration. We measured immunoglobin G (IgG) antibodies to HSV-1, CMV, EBV, VZV, and TOX. After adjusting for sociodemographics, body mass index, comorbidities, household income, and household size, there were no associations between common infections and any sleep outcomes ; however, we observed interactions with race. Among White participants only, CMV seropositivity was associated with a lower odds of insomnia symptoms (OR = 0.26, 95% CI 0.10, 0.69) and TOX seropositivity was associated with shorter sleep duration (OR = 0.65, 95% CI 0.09, 1.21). Further research is needed to investigate mechanisms that may account for associations of CMV and TOX with sleep among White but not minoritized participants.

